# Associations between Dietary Animal and Plant Protein Intake and Cardiometabolic Risk Factors—A Cross-Sectional Study in China Health and Nutrition Survey

**DOI:** 10.3390/nu13020336

**Published:** 2021-01-23

**Authors:** Shuangli Meng, Zhixin Cui, Minjuan Li, Ting Li, Feng Wu, Tong Kang, Huicui Meng

**Affiliations:** 1 School of Public Health (Shenzhen), Sun Yat-Sen University, Shenzhen 518106, China; mengshl@mail2.sysu.edu.cn (S.M.); cuizhx3@mail2.sysu.edu.cn (Z.C.); limj36@mail2.sysu.edu.cn (M.L.); liting66@mail2.sysu.edu.cn (T.L.); wufeng29@mail2.sysu.edu.cn (F.W.); kangt7@mail2.sysu.edu.cn (T.K.); 2 Guangdong Provincial Key Laboratory of Food, Nutrition and Health, Guangzhou 510080, China; 3 Guangdong Province Engineering Laboratory for Nutrition Translation, Guangzhou 510080, China

**Keywords:** animal protein, plant protein, cardiometabolic risk factor, lipid and lipoprotein profiles, glucose homeostasis biomarker, low-grade chronic inflammatory biomarker, uric acid

## Abstract

Available data investigating the associations between dietary animal and plant protein intakes and cardiometabolic risk factors (CMRFs) among populations with habitual plant-based diets are heterogenous and limited in scope. The current study was to assess the associations between dietary animal and plant protein intakes and CMRFs, including lipid and lipoprotein profiles, glucose homeostasis biomarkers, low-grade chronic inflammatory biomarker and uric acid in Chinese adults. Data of 7886 apparently healthy adults were extracted from the China Health and Nutrition Survey 2009. Dietary protein (total, animal and plant) intakes were assessed with three consecutive 24 h dietary recalls, and CMRFs were measured with standard laboratory methods. Substituting 5% of energy intake from animal protein for carbohydrates was positively associated with total cholesterol, low-density lipoprotein cholesterol (LDL-C), non-high-density lipoprotein cholesterol (non-HDL-C) and uric acid (all *p* < 0.05). Substituting 5% of energy intake from plant protein for carbohydrates was inversely associated with non-HDL-C and LDL-C:HDL-C ratio, and positively associated with HDL-C and glycated hemoglobin (all *p* < 0.05). Some of these associations varied in subgroup analyses by BMI, sex, age or region. There were no significant associations between animal or plant protein intakes and high-sensitivity C-reactive protein. The public health implication of these findings requires further investigation.

## 1. Introduction

Cardiometabolic disorders, including cardiovascular diseases (CVD), type 2 diabetes and metabolic syndrome, are the leading causes of death globally [1]. The prevalence of these disorders has been increasing dramatically over the decades, which has become a worldwide public health problem [2,3]. Cardiometabolic risk is largely attributable to a lack of adherence to healthy lifestyle behaviors, such as healthy eating habits and regular physical activity [4]. Results from observational studies have consistently reported positive associations between dietary animal protein intake and risk of type 2 diabetes [5], coronary heart disease [6,7] and ischemic heart disease [8] and CVD-specific mortality [9,10,11], and inverse associations between dietary plant protein intake and cardiometabolic disorder outcomes [5,8,9,10]. Although potential mechanisms underpinning these associations have been attributed to the effects of animal or plant protein intake on traditional cardiometabolic risk factors (CMRFs), including lipid and lipoprotein profiles, glucose homeostasis biomarkers and low-grade chronic inflammation, and emerging risk factor serum uric acid or hyperuricemia [12], data supporting these assumptions are strikingly limited and inconsistent.

Several studies have reported that dietary animal protein intake was positively associated with concentrations of total cholesterol (TC) [13] and low-density lipoprotein cholesterol (LDL-C) [14]. Results from interventional studies have also demonstrated that plant protein supplementation results in more favorable serum concentrations of high-density lipoprotein cholesterol (HDL-C), TC, triglycerides (TG), LDL-C, and non-HDL-C compared with animal protein [15,16]. In contrast, other observational studies have found no significant associations between animal or plant protein intake and lipid profiles [17,18]. In addition, available data for the associations between dietary animal and plant protein intake and glucose homeostasis biomarkers [17,19], low-grade inflammatory biomarkers [20,21], and uric acid [20,22] also have heterogeneous findings. Research suggests that the inconsistency may be partially driven by the differential background dietary patterns of study participants [23]. The majority of the available studies have focused on populations with habitual animal-based dietary patterns, such as the western diet. There are strikingly incomplete data on the associations between dietary animal and plant protein intake and CMRFs in Asian populations, who usually follow plant-based dietary patterns, while bearing an increasing burden of dyslipidemia, hyperglycemia, and hyperuricemia [24]. Relative comparisons of associations between substituting animal versus plant protein for carbohydrate and CMRFs are also unclear. This lack of information undermines efforts to provide more precise dietary guidance aimed to reduce cardiometabolic risk in Asian populations.

The primary aim of the present study was to assess the associations between an isocaloric substitution of dietary animal or plant protein intake for carbohydrate and CMRFs, including lipid and lipoprotein profiles, glucose homeostasis biomarkers, low-grade chronic inflammatory biomarkers, and uric acid, in a nationwide cohort of Chinese adults. The secondary and exploratory aim was to assess the associations in subgroups by body mass index (BMI), sex, age and region. We hypothesized that an isocaloric substitution of animal protein for carbohydrates was associated with unfavorable CMRFs, and plant protein had opposite associations.

## 2. Subjects and Methods 

### 2.1. Study Population

The China Health and Nutrition Survey (CHNS) is an ongoing large-scale longitudinal study aimed at examining the effects of social and economic transformation on the health and nutritional status of Chinese residents. The CHNS was initiated in 1989, and ten rounds of surveys have been completed in 1989, 1991, 1993, 1997, 2000, 2004, 2006, 2009, 2011 and 2015. The same cohort of populations in the representative areas of nine provinces (Liaoning, Heilongjiang, Shandong, Henan, Jiangsu, Hubei, Hunan, Guizhou, and Guangxi) were followed up in the ten rounds of surveys. Participants from three mega cities (Beijing, Shanghai and Chongqing) have joined this survey since 2011, and participants from three additional provinces (Yunnan, Shaanxi and Zhejiang) have joined since 2015 [25,26]. Over 30,000 participants from 15 provinces were recruited with the use of a multistage random cluster process. The detailed information of the purpose and design of the CHNS has been described previously [26]. The study was conducted according to the Declaration of Helsinki guidelines. All study procedures were approved by the University of North Carolina at Chapel Hill and the National Institute for Nutrition and Health at the Chinese Center for Disease Control and Prevention (under project identification code provided previously [27,28]), and written informed consent was obtained from all participants.

In the current investigation, our data were extracted from the 2009 wave of the CHNS. We excluded participants who were diagnosed with diabetes, myocardial infarction, stroke, or tumor, or who were currently taking medicines that may affect glucose, lipid metabolism, or uric acid concentrations (*n* = 427), or who were pregnant (*n* = 59) or breast-feeding (*n* = 42). We also excluded participants who were aged below 18 years old (*n* = 1634), who had missing values in all dietary records (*n* = 569), circulating biomarker assessments (*n* = 1420), or who had an implausible total calorie intake (<800 or >4200 kcal/day for men and <500 or >3500 kcal/day for women, *n* = 117) [29]. A total of 7886 participants were included (3690 men and 4196 women) in the final analysis (Figure S1).

### 2.2. Dietary Assessment

Dietary intake data were collected with the use of three consecutive 24 h dietary recalls at the individual level and a food inventory method at the household level [30]. Participants provided information on the type, amount, preparation method, and eating location for every single food and beverage consumed within the past 24 h for three days, including two weekdays and one weekend, under the instruction of trained interviewers [30]. The amount of food consumption was confirmed by a food weighing method at the household level during the same three-day period [30].

Nutrient intakes were calculated by multiplying the amount of consumption by the nutrient content of the edible portions of each food. Nutrient intakes of all foods were summed for each day, and the mean of three-day intakes was calculated to estimate the average daily nutrient intakes. The nutrient contents of each food were obtained from the China Food Composition Tables [31,32,33]. Protein intakes from both animal and plant sources were calculated. Animal protein sources included red meat, white meat, offal, fish, seafood, eggs, and dairy foods. Plant protein sources included legumes, rice, wheaten foods, coarse grains, tubers, nuts, seeds, fruits, vegetables, fungi, and algae. Other protein sources (approximately 0.47% of total energy intake) were foods that could not be obviously classified as either animal or plant protein sources (e.g., chocolate) and were not classified as either of them [34]. The animal to plant protein ratio was also calculated, with a higher ratio indicating a higher animal-based dietary pattern. All nutrient intakes were adjusted for total energy intake by the regression residual method [35], and each nutrient, except for fiber, was expressed as the percentage of total energy.

### 2.3. Assessment of CMRF

Venous blood samples were collected from each participant following an overnight fasting for 8–12 h. Serum samples were separated by centrifugation at 3000× *g* for 15 min, and were immediately stored at −86 °C for subsequent laboratory analysis. All samples were analyzed in a verified national central lab in Beijing (medical laboratory accreditation certificate ISO 15189:2007) under strict quality control [36]. Serum concentrations of TG, TC, HDL-C, LDL-C, lipoprotein (a), high-sensitivity C-reactive protein (hsCRP), glucose, and uric acid were measured on the Hitachi 7600 automated analyzer (Hitachi Inc., Tokyo, Japan), with corresponding reagents [(Denka Seiken Co., Ltd., Tokyo, Japan) for lipoprotein (a) and hsCRP, (Randox Laboratories Ltd., Crumlin, UK) for glucose and uric acid, and (Kyowa Medex Co., Ltd., Tokyo, Japan) for others] [36]. Serum insulin concentrations were determined via radioimmunology on a Gamma counter XH-6020 (North Institute of Bio-Tech, Beijing, China). Whole blood glycated hemoglobin A1c (HbA1c) was measured via a high-performance liquid chromatography system (HLC-723 G7; Tosoh Corporation, Tokyo, Japan) [36]. The non-HDL-C concentrations and the TC:HDL-C and LDL-C:HDL-C ratios were calculated as follows:

non-HDL-C = TC − HDL-C,(1)

TC:HDL-C = TC/HDL-C,(2)

LDL-C:HDL-C = LDL-C/HDL-C,(3)

Homeostasis model assessment of insulin resistance (HOMA-IR) was calculated using the Matthews formula [37]:

HOMA-IR = (fasting insulin × fasting glucose)/22.5(4)

### 2.4. Assessment of Other Covariates

Data on the sociodemographic, anthropometric, and lifestyle characteristics of participants were collected with validated questionnaires by trained interviewers [36]. The height and weight of participants were measured without shoes and in light clothing using calibrated instruments. The height was accurate to 0.1 cm and the weight was accurate to 0.1 kg. The BMI was computed as the ratio of weight (kg) to the square of height (m^2^) [36]. According to the guidelines for the prevention and control of overweightness and obesity in Chinese adults [38], the BMI was classified as underweight (<18.5 kg/m^2^), normal weight (18.5–23.9 kg/m^2^), overweight (24–27.9 kg/m^2^) or obese (≥28 kg/m^2^). Waist circumference and hip circumference were measured using an inelastic tape to the nearest 0.1 cm. Waist circumference was measured at a midpoint between the bottom of the rib cage and the top of the iliac crest at the end of exhalation. Hip circumference was measured at the level of maximal gluteal protrusion. The waist to hip ratio was computed as the waist circumference divided by the hip circumference [39]. Systolic blood pressure (SBP) and diastolic blood pressure (DBP) were measured on the right arm after a 10 min seated rest by trained examiners using a mercury sphygmomanometer. Measures were repeated for three times with an interval of 3–5 min [36,40], and the average of the three measurements was used in analysis. Physical activity information was collected with a validated self-reported questionnaire, and metabolic equivalent-hours/week (MET-h/week) was assessed based on the time and intensity of occupational, household, and leisure time and transportation activities [39,41]. Region was divided into northern or southern China geographically by the Qinling Mountains–Huaihe River line [42]. Northern regions included Liaoning, Heilongjiang, Shandong and Henan provinces, and southern regions included Jiangsu, Hubei, Hunan, Guizhou and Guangxi provinces.

### 2.5. Statistical Analysis

All statistical analyses were performed with SAS 9.4 (SAS Institute, Cary, NC, USA). Sociodemographic, anthropometric, and lifestyle characteristics of participants were presented on the basis of quintiles of animal and plant protein intakes. Continuous variables were presented as mean ± standard deviation (SD), and categorical variables were presented as *n* (%). Linear regression models were used to investigate the associations between total, animal or plant protein intake (per 5% energy, which was the original variable divided by five) and CMRFs (lipid and lipoprotein profiles, glucose homeostasis biomarkers, low-grade inflammatory biomarkers and uric acid). Model 1 was a simple linear regression model. Model 2 was a multiple linear regression model, in which the data were adjusted for potential confounders, including age (<50 years, 50–54 years, 55–59 years, 60–64 years or ≥65 years), sex (women or men), BMI (<18.5 kg/m^2^, 18.5–23.9 kg/m^2^, 24–27.9 kg/m^2^ or ≥28 kg/m^2^), urban index (low, medium or high), region [northern (Liaoning, Heilongjiang, Shandong and Henan) or southern (Jiangsu, Hubei, Hunan, Guizhou and Guangxi)], education level [primary (primary school or lower), middle (middle school) or high (high school or above)], alcohol intake [yes (at least once a month) or no], current smoker [yes (at least once a month) or no], physical activity (low, medium or high based on tertiles of MET-h/week), blood pressure [normal (SBP < 120 mm Hg and DBP < 80 mm Hg), pre-hypertension (SBP of 120–139 mm Hg or DBP of 80–89 mm Hg) or hypertension (SBP ≥ 140 mm Hg or DBP ≥ 90 mm Hg)] and dietary variables, including total energy (continuous as kcal/day), fiber (continuous as g/day), and cholesterol, saturated fatty acids (SFA), monounsaturated fatty acids (MUFA), polyunsaturated fatty acids (PUFA) and other fatty acids (all continuous as % of total energy). Models with animal protein included plant protein as an additional confounder, and models with plant protein included animal protein as an additional confounder. In Model 2, carbohydrate intake was not included as a confounder in order to construct as an isocaloric substitution model, in which 5% energy from total, animal or plant protein was substituted for carbohydrates. In subsequent analyses, data were divided into subgroups on the basis of participants’ BMI (<24 kg/m^2^ or ≥24 kg/m^2^), sex (women or men), age (<60 years or ≥60 years), and region (northern or southern), and the associations were assessed in each subgroup with the same confounders in Model 2, except for BMI, sex, age or region, respectively. Models 1 and 2 and subgroup analyses were also conducted to assess the associations between dietary animal to plant protein intake and CMRFs. A two-tailed *p* < 0.05 was considered statistically significant.

## 3. Results

### 3.1. Sociodemographic, Anthropometric and Lifestyle Characteristics of Participants

The sociodemographic, anthropometric and lifestyle characteristics of the study participants according to quintiles (Q) of energy-adjusted animal and plant protein intakes are presented in Table 1. The mean age of the participants was 50 years, 53.2% were females, and the mean BMI was 23.3 kg/m^2^. The mean percentage of total energy from total, animal and plant protein were 14.4%, 4.7% and 9.2%, respectively. The dietary animal to plant protein ratio of the participants was approximately 0.6, indicating a plant-based dietary pattern among the study population (Table 1). The average age, BMI, waist to hip ratio and blood pressures, as well as the percentage of current smokers of the participants, were similar across the quintiles of animal or plant protein intakes (Table 1). The mean percentage of total energy from SFA, MUFA, PUFA and cholesterol were 4.9%, 7.1%, 2.3% and 0.1%, respectively. In general, participants with higher intakes of animal protein were more likely to be physically inactive, drink alcohol, receive higher education, reside in areas with a high urban index and in southern provinces, and have higher intakes of dietary fat and cholesterol and lower intakes of carbohydrates and fiber in comparison to those who had lower intakes of animal protein. A reverse tendency was observed among participants who had higher intakes of plant protein. The mean values of the serum CMRF concentrations were in the normal range based on the established criteria [43].

### 3.2. Food Sources of Total, Animal, and Plant Protein

The top three food sources of dietary total protein were grains, red meat and legumes, which collectively contributed more than 70% of the total protein intake (Table 2). The main sources of animal protein consisted of red meat and eggs. Grains were the principal source of plant protein, followed by legumes, fruits and vegetables. 

### 3.3. Associations Between Dietary Total, Animal, and Plant Protein Intakes and CMRFs

In unadjusted models (Model 1), dietary total protein intake was associated with fasting serum concentrations of TG, TC, LDL-C, non-HDL-C and uric acid, as well as TC:HDL-C and LDL-C:HDL-C ratios (all *p* < 0.01) (Table 3). In fully adjusted models (Model 2), substituting 5% of energy intake from the total protein for carbohydrates was associated with increases in serum concentrations of TC (β = 0.05 mmol/L, *p* = 0.03), HDL-C (β = 0.02 mmol/L, *p* = 0.04) and uric acid (β = 7.44 μmol/L, *p* < 0.01) (Table 3). There were no significant associations between total protein intake and other CMRFs.

In the unadjusted models, dietary animal protein intake was associated with fasting concentrations of TG, TC, LDL-C, non-HDL-C, glucose, HbA1c and uric acid, as well as TC:HDL-C and LDL-C:HDL-C ratios (all *p* < 0.05) (Table 3). In fully-adjusted models, substituting 5% of energy intake from animal protein for carbohydrates was associated with increases in serum concentrations of TC (β = 0.09 mmol/L, *p* < 0.01), LDL-C (β = 0.05 mmol/L, *p* = 0.04), non-HDL-C (β = 0.08 mmol/L, *p* < 0.01) and uric acid (β = 15.32 μmol/L, *p* < 0.01) (Table 3). There were no significant associations between animal protein intake and glucose homeostasis and low-grade inflammatory biomarkers. 

In the unadjusted models, dietary plant protein intake was associated with fasting concentrations of TC, LDL-C, non-HDL-C, glucose, HbA1c and uric acid and LDL-C:HDL-C ratio (all *p* < 0.01) (Table 3). In the fully-adjusted models, substituting 5% of energy intake from plant protein for carbohydrates was inversely associated with serum concentration of non-HDL-C (β = −0.06 mmol/L, *p* < 0.05) and LDL-C:HDL-C ratio (β = −0.06, *p* = 0.01), and positively associated with serum concentrations of HDL-C (β = 0.02 mmol/L, *p* = 0.03) and HbA1c (β = 0.07%, *p* < 0.01) (Table 3). There were no significant associations between plant protein intake and other CMRFs. 

In the unadjusted models, the animal to plant protein ratio was associated with fasting concentrations of TG, TC, LDL-C, non-HDL-C, HbA1c and uric acid, as well as TC: LDL-C and LDL-C:HDL-C ratios (all *p* < 0.05) (Table S1). In the fully-adjusted models, the animal to plant protein ratio was positively associated with serum concentrations of TC (β = 0.10 mmol/L, *p* < 0.01), LDL-C (β = 0.09 mmol/L, *p* < 0.01), non-HDL-C (β = 0.08 mmol/L, *p* = 0.01) and uric acid (β = 15.53 μmol/L, *p* < 0.01), as well as the LDL-C:HDL-C ratio (β = 0.06, *p* = 0.01), and inversely associated with serum concentrations of HbA1c (β = 0.06%, *p* = 0.01) (Table S1). There were no significant associations between the animal to plant protein ratio and other CMRFs.

### 3.4. Subgroup Analyses on the Basis of BMI, Sex, Age, and Region

Further investigation showed that, in participants with a BMI < 24 kg/m^2^ (underweight or normal weight), substituting 5% of energy intake from total protein for carbohydrates was associated with increases in serum concentrations of TC (β = 0.08 mmol/L, *p* = 0.01), HDL-C (β = 0.02 mmol/L, *p* = 0.04) and uric acid (β = 8.35 μmol/L, *p* < 0.01) (Table 4). Substituting 5% of energy intake from animal protein for carbohydrates was associated with increases in serum concentrations of TG (β = 0.08 mmol/L, *p* = 0.02), TC (β = 0.12 mmol/L, *p* < 0.01), LDL-C (β = 0.08 mmol/L, *p* = 0.02), non-HDL-C (β = 0.10 mmol/L, *p* < 0.01) and uric acid (β = 15.82 μmol/L, *p* < 0.01), as well as the TC:HDL-C ratio (β = 0.07, *p* = 0.04) (Table 4). Substituting 5% of energy intake from plant protein for carbohydrates was inversely associated with TC:HDL-C (β = −0.10, *p* = 0.02) and LDL-C:HDL-C (β = −0.11, *p* < 0.01) ratios, and positively associated with serum concentrations of HDL-C (β = 0.04 mmol/L, *p* = 0.01) and HbA1c (β = 0.06%, *p* < 0.05) (Table 4). The animal to plant protein ratio was associated with increases in serum concentrations of TG (β = 0.08 mmol/L, *p* = 0.03), TC (β = 0.12 mmol/L, *p* < 0.01), LDL-C (β = 0.11 mmol/L, *p* < 0.01), non-HDL-C (β = 0.12 mmol/L, *p* < 0.01) and uric acid (β = 15.76 μmol/L, *p* < 0.01), as well as TC:HDL-C (β = 0.11, *p* < 0.01) and LDL-C:HDL-C (β = 0.09, *p* < 0.01) ratios (Table S2).

In participants with a BMI ≥ 24 kg/m^2^ (overweight or obese), there were no significant associations between dietary total protein intake and any CMRF (Table 4). Substituting 5% of energy intake from animal protein for carbohydrates was only associated with an increase in serum concentration of uric acid (β = 14.91 μmol/L, *p* < 0.01), and substituting 5% of energy intake from plant protein for carbohydrates was only associated with an increase in serum concentration of HbA1c (β = 0.08%, *p* < 0.05) (Table 4). The animal to plant protein ratio was positively associated with serum concentrations of uric acid (β = 14.96 μmol/L, *p* < 0.01), and inversely associated with serum concentrations of glucose (β = −0.16 mmol/L, *p* = 0.03) and HbA1c (β = −0.10%, *p* = 0.03) (Table S2).

In women, substituting 5% of energy intake from total protein for carbohydrates was only associated with an increase in serum concentration of uric acid (β = 7.25 μmol/L, *p* < 0.01) (Table 5). Substituting 5% of energy intake from animal protein for carbohydrates was associated with increases in serum concentrations of TC (β = 0.08 mmol/L, *p* = 0.04) and uric acid (β = 14.81 μmol/L, *p* < 0.01) (Table 5). There were no significant associations between dietary plant protein intake and CMRFs (Table 5). The animal to plant protein ratio was only associated with an increase in serum concentration of uric acid (β = 15.70 μmol/L, *p* < 0.01) (Table S3).

In men, substituting 5% of energy intake from total protein for carbohydrates was only associated with an increase in serum concentration of HDL-C (β = 0.03 mmol/L, *p* = 0.03) (Table 5). Substituting 5% of energy intake from animal protein for carbohydrates was associated with increases in serum concentrations of TC (β = 0.09 mmol/L, *p* = 0.02) and uric acid (β = 14.65 μmol/L, *p* < 0.01) (Table 5). Substituting 5% of energy intake from plant protein for carbohydrates was inversely associated with serum concentrations of TC (β = −0.09 mmol/L, *p* < 0.05), LDL-C (β = −0.17 mmol/L, *p* < 0.01) and non-HDL-C (β = −0.14 mmol/L, *p* < 0.01), as well as TC:HDL-C (β = −0.11, *p* < 0.05) and LDL-C:HDL-C (β = −0.18, *p* < 0.01) ratios, and positively associated with serum concentrations of HDL-C (β = 0.04 mmol/L, *p* = 0.01) and HbA1c (β = 0.08%, *p* = 0.03) (Table 5). The animal to plant protein ratio was positively associated with serum concentrations of TC (β = 0.10 mmol/L, *p* = 0.01), LDL-C (β = 0.12 mmol/L, *p* < 0.01) and uric acid (β = 14.13 μmol/L, *p* < 0.01), as well as the LDL-C:HDL-C ratio (β = 0.08, *p* = 0.03) (Table S3).

In young and middle-aged participants, substituting 5% of energy intake from total protein for carbohydrates was associated with increases in serum concentrations of HbA1c (β = 0.05%, *p* < 0.05) and uric acid (β = 7.62 μmol/L, *p* < 0.01) (Table 6). Substituting 5% of energy intake from animal protein for carbohydrates was associated with increases in serum concentrations of TC (β = 0.09 mmol/L, *p* < 0.01), non-HDL-C (β = 0.08 mmol/L, *p* = 0.01) and uric acid (β = 15.55 μmol/L, *p* < 0.01), as well as the TC:HDL-C ratio (β = 0.08, *p* = 0.04) (Table 6). Substituting 5% of energy intake from plant protein for carbohydrates was inversely associated with the serum LDL-C:HDL-C ratio (β = −0.07, *p* = 0.03) and positively associated with serum concentration of HbA1c (β = 0.09%, *p* < 0.01) (Table 6). The animal to plant protein ratio was associated with increases in serum concentration of uric acid (β = 14.78 μmol/L, *p* < 0.01) and LDL-C:HDL-C ratio (β = 0.07, *p* = 0.01) (Table S4).

In elderly participants, there were no significant associations between dietary total and plant protein intakes and CMRFs (Table 6). Substituting 5% of energy intake from animal protein for carbohydrates was positively associated with serum concentration of uric acid (β = 15.21 μmol/L, *p* < 0.01), and inversely associated with serum concentration of HbA1c (β = −0.17%, *p* < 0.01) (Table 6). The animal to plant protein ratio was positively associated with serum concentrations of TC (β = 0.17 mmol/L, *p* = 0.01) and uric acid (β = 19.12 μmol/L, *p* < 0.01), and inversely associated with serum concentration of HbA1c (β = −0.20%, *p* < 0.01) (Table S4).

In participants who resided in North China, substituting 5% of energy intake from total protein for carbohydrates was only associated with an increase in the serum concentration of TC (β = 0.12 mmol/L, *p* < 0.01) (Table 7). Substituting 5% of energy intake from animal protein for carbohydrates was associated with increases in serum concentrations of TC (β = 0.14 mmol/L, *p* < 0.01), non-HDL-C (β = 0.11 mmol/L, *p* = 0.04) and uric acid (β = 21.38 μmol/L, *p* < 0.01), and decreases in serum concentrations of glucose (β = −0.18 mmol/L, *p* = 0.01) and HbA1c (β = −0.16%, *p* < 0.01) (Table 7). Substituting 5% of energy intake from plant protein for carbohydrates was only inversely associated with serum concentration of uric acid (β = −9.23 μmol/L, *p* = 0.02) (Table 7). The animal to plant protein ratio was positively associated with serum concentrations of TC (β = 0.12 mmol/L, *p* = 0.02) and uric acid (β = 24.82 μmol/L, *p* < 0.01), and inversely associated with the serum concentration of HbA1c (β = −0.15%, *p* < 0.01) (Table S5).

In participants who resided in South China, substituting 5% of energy intake from total protein for carbohydrates was associated with increases in serum concentrations of HbA1c (β = 0.06%, *p* < 0.01) and uric acid (β = 10.78 μmol/L, *p* < 0.01) (Table 7). Substituting 5% of energy intake from animal protein for carbohydrates was associated with increases in serum concentrations of TC (β = 0.10 mmol/L, *p* < 0.01), LDL-C (β = 0.07 mmol/L, *p* = 0.04), non-HDL-C (β = 0.10 mmol/L, *p* < 0.01) and uric acid (β = 14.08 μmol/L, *p* < 0.01) (Table 7). Substituting 5% of energy intake from plant protein for carbohydrates was inversely associated with the serum LDL-C:HDL-C ratio (β = −0.10, *p* < 0.01), and positively associated with serum concentrations of HDL-C (β = 0.04 mmol/L, *p* = 0.01) and HbA1c (β = 0.17%, *p* < 0.01) (Table 7). The animal to plant protein ratio was positively associated with serum concentrations of TC (β = 0.09 mmol/L, *p* = 0.01), LDL-C (β = 0.09 mmol/L, *p* = 0.01), non-HDL-C (β = 0.08 mmol/L, *p* = 0.03) and uric acid (β = 11.07 μmol/L, *p* < 0.01), as well as the LDL-C:HDL-C ratio (β = 0.06, *p* = 0.03) (Table S5).

## 4. Discussion

Several large cohorts have confirmed that total and animal protein intake are associated with an increased risk for cardiovascular disease and type 2 diabetes, while plant protein intake has potentially favorable effects on cardiometabolic health [23,44]. However, there is strikingly limited data on the associations between animal and plant protein intake and lipid and lipoprotein profiles, glucose homeostasis biomarkers, low-grade chronic inflammatory biomarkers and uric acid, all of which collectively contribute to increased cardiometabolic risk. The unique aspect of our study is that we targeted a population following habitual plant-based dietary patterns. The results indicated that dietary animal protein intake was positively associated with fasting serum concentrations of TC, LDL-C, non-HDL-C and uric acid, while plant protein intake was inversely associated with non-HDL-C concentrations and LDL-C:HDL-C ratio and positively associated with HDL-C and HbA1c concentrations in all participants. Consistent with prior studies that reported data for hsCRP [21,45,46], our results found no significant associations between animal or plant protein intake and hsCRP. There were also no significant associations between animal or plant protein intake and glucose homeostasis biomarkers, except for an unexpected positive association between plant protein intake and HbA1c. Subgroup analyses demonstrated that these associations may vary by BMI, sex, age, or region. 

Dietary animal protein intake was positively associated with fasting serum concentrations of TC, LDL-C and non-HDL-C, while plant protein intake was inversely associated with non-HDL-C concentrations and LDL-C:HDL-C ratio and positively associated with HDL-C concentrations in all participants. These findings are consistent with much of the data from both observational [13,14,47] and interventional [15,16] studies reported previously. The potential underlying mechanisms responsible for the differential associations between animal versus plant protein and lipid and lipoprotein profiles may be attributable, in part, to the different amino acid compositions of animal and plant proteins [48]. Animal protein provides greater amounts of essential amino acids, such as methionine and lysine, which have been reported to promote hypercholesterolemia in experimental animals [49]. Conversely, plant protein is rich in nonessential amino acids, including arginine, which may affect hypercholesterolemia via the L-arginine-nitric oxide pathway [50]. Another proposed mechanism is alteration in gut microbial composition in response to animal compared to plant protein supplementation in both human participants [51] and experimental animals [52], concurrently with changes in lipid metabolism. In addition, certain amino acids in animal protein, such as carnitine, can be metabolized to trimethylamine-N-oxide (TMAO) by gut microbiota and hepatic metabolism [44]. Elevated circulating TMAO concentrations have consistently been shown to be correlated with an adverse cardiovascular event [44], possibly via alterations in reverse cholesterol transport, bile acid composition and cholesterol metabolism [44,53]. Gut microbiota may play an important role in cholesterol metabolism through secondary bile acid synthesis, short-chain fatty acids and bacteria-derived pro-inflammatory mediators, such as lipopolysaccharide [54,55], and hence a comprehensive analysis of gut microbial composition and related metabolites in response to animal and plant protein is needed to explore this potential mechanism. 

Dietary animal or plant protein were not significantly associated with fasting concentrations of glucose and insulin or HOMA-IR score when all participants were analyzed together. However, subgroup analysis by region revealed that animal protein intake was inversely associated with fasting glucose concentrations in participants who resided in North China. Similar to our findings, a study in middle-aged and elderly Finnish men has demonstrated associations between a higher consumption of egg, which is a major source of animal protein, and lower fasting glucose concentrations [21]. In addition, a German randomized controlled trial has reported a significant reduction in fasting glucose concentration following six-week consumption of a diet enriched in animal protein, but not plant protein [20]. Consumption of animal protein may induce a postprandial hypoglycemic response, which is mediated via its stimulatory effect on the release of gut hormones, such as glucagon-like peptide-1, gastric inhibitory polypeptides and cholecystokinin, and the reduction of gastric-emptying rates [56,57]. Hypoglycemic response of protein has also been attributed in part to the stimulation of insulin secretion from pancreatic β cells [56,57]. However, there was no significant association between animal protein intake and fasting insulin concentrations in our participants. The discrepancies in the associations between animal protein intake and fasting glucose concentrations between northern and southern participants were unclear, and may be driven by the differences in background dietary patterns between regions [58]. Plant protein intake was positively associated with HbA1c, and subgroup analysis revealed that this association existed in male participants, young and middle-aged adults and people who resided in South China. These findings were somewhat unexpected, because no or inverse association has been previously reported [20,59]. The reason for the inconsistent results is not obvious.

Higher dietary total protein intake was associated with increased uric acid concentrations, and this association was mainly due to the positive association between animal protein intake and uric acid concentrations, which is consistent with results from previous studies [22,51,60]. The potential underlying mechanisms of the positive associations between animal protein and uric acid levels may be attributable to the effects of amino acids on purine synthesis [60] and/or the exogenous purine from foods enriched in animal protein [60,61,62]. In contrast to our findings, a randomized controlled trial conducted in elderly type 2 diabetic patients has reported that animal protein supplementation results in reduction of uric acid [20]. The reason for the discordant results may be due to differences in study participants, as the participants in our study were apparently healthy. We also observed inverse associations between plant protein intake and uric acid levels in participants from North China, indicating possible the beneficial effects of plant protein consumption on regulating uric acid levels. Mechanisms to substantiate this beneficial effect have not been explored. The lack of a significant association between animal or plant protein intake and hsCRP concentration is consistent with prior reports [21,45,46].

There are several strengths in this study. Collections of data on sociodemographic, anthropometric and lifestyle characteristics and dietary intake of participants and laboratory analyses of CMRFs were under strict quality controls. The targeted populated was following habitual plant-based dietary patterns, and the associations between animal and protein intake and CMRFs in these participants are most likely to differ from individuals following habitual animal-based dietary patterns, such as the western diet. In addition, a wider array of CMRFs was investigated than previously reported. Some limitations of the study need to be addressed. Our study is a cross-sectional study, and was unable to establish causal relationship and explore mechanisms underpinning the associations between animal or plant protein and certain CMRFs. As is the case with all observational studies, there may be other residual or unmeasured confounders in addition to the many confounders adjusted for in the current analyses.

## 5. Conclusions

In conclusion, dietary animal protein intake was positively associated with fasting serum concentrations of TC, LDL-C, non-HDL-C and uric acid, while plant protein intake was inversely associated with non-HDL-C concentrations and LDL-C:HDL-C ratio and positively associated with HDL-C and HbA1c concentrations in all participants. There were no significant associations between animal or plant protein intake and fasting concentrations of glucose, insulin or hsCRP or HOMA-IR score. Subgroup analyses demonstrated that these associations may vary by BMI, sex, age or region. Further studies are required to confirm the causal relationship between animal and plant protein and CMRFs in Chinese populations and public health implications of these findings via randomized controlled trials.

## Figures and Tables

**Table 1:** Sociodemographic, anthropometric and lifestyle characteristics of 7886 Chinese adults who participated in the China Health and Nutrition Survey 2009 by quintiles of energy-adjusted animal or plant protein intakes. ^1^

Characteristics	All Participants	Animal Protein			Plant Protein		
		Q1	Q3	Q5	Q1	Q3	Q5
Median of each quintile,% of total energy	-	0.8	4.2	8.9	6.3	8.9	12.3
Number or participants, *n*	7886	1571	1569	1631	1553	1591	1656
Age, y	50 ± 15	52 ± 15	50 ± 15	48 ± 15	49 ± 15	50 ± 15	51 ± 14
Sex, *n* (%)							
Women	4196 (53.2)	869 (55.3)	860 (54.8)	822 (50.4)	816 (52.5)	837 (52.6)	902 (54.5)
Men	3690 (46.8)	702 (44.7)	709 (45.2)	809 (49.6)	737 (47.5)	754 (47.4)	754 (45.5)
BMI, kg/m^2^	23.3 ± 3.4	23.3 ± 3.3	23.2 ± 3.4	23.2 ± 3.5	23.1 ± 3.3	23.3 ± 3.5	23.6 ± 3.4
Waist to hip ratio	0.9 ± 0.1	0.9 ± 0.1	0.9 ± 0.1	0.9 ± 0.1	0.9 ± 0.1	0.9 ± 0.1	0.9 ± 0.1
Urban index, *n* (%)							
Low	2623 (33.3)	1022 (65.1)	435 (27.7)	212 (13.0)	249 (16.0)	465 (29.2)	889 (53.7)
Medium	2600 (33.0)	422 (26.9)	641 (40.9)	427 (26.2)	548 (35.3)	562 (35.3)	426 (25.7)
High	2663 (33.8)	127 (8.1)	493 (31.4)	992 (60.8)	756 (48.7)	564 (35.4)	341 (20.6)
Region, *n* (%)							
Northern	3311 (42.0)	1012 (64.4)	619 (39.5)	396 (24.3)	435 (28.0)	646 (40.6)	991 (59.8)
Southern	4575 (58.0)	559 (35.6)	950 (60.5)	1235 (75.7)	1118 (72.0)	945 (59.4)	665 (40.2)
Higher education, *n* (%)	1901 (24.1)	187 (11.9)	348 (22.2)	625 (38.3)	484 (31.2)	398 (25.0)	285 (17.2)
Alcohol intake, *n* (%)	2601 (33.0)	451 (28.7)	499 (31.8)	647 (39.7)	540 (34.8)	541 (34.0)	519 (31.3)
Current smokers, *n* (%)	2214 (28.1)	466 (29.7)	424 (27.0)	490 (30.0)	441 (28.4)	441 (27.7)	453 (27.4)
Physical activity level, MET-h/week	69.0 ± 100.1	97.4 ± 118.5	66.5 ± 97.3	46.3 ± 81.3	53.7 ± 86.7	67.8 ± 99.9	81.3 ± 110.4
SBP, mmHg	124.3 ± 18.7	124.7 ± 18.4	125.0 ± 19.3	123.0 ± 18.2	123.6 ± 19.0	124.3 ± 18.6	125.7 ± 17.6
DBP, mmHg	80.1 ± 11.1	81.1 ± 10.8	79.6 ± 11.2	79.7 ± 11.2	79.3 ± 11.1	80.1 ± 11.4	81.7 ± 10.8
Dietary intakes							
Total energy, kcal/day	1729.7 ± 14.5	1729.2 ± 9.6	1729.7 ± 13.6	1731.0 ± 19.7	1730.5 ± 22.2	1729.7 ± 11.4	1728.6 ± 10.4
Protein, % of total energy	14.4 ± 3.0	12.4 ± 2.1	13.8 ± 2.1	17.8 ± 2.8	14.3 ± 3.3	14.1 ± 2.7	15.4 ± 3.1
Animal protein,% of total energy	4.7 ± 3.3	0.8 ± 0.6	4.2 ± 0.5	9.7 ± 2.5	7.2 ± 3.5	4.8 ± 2.7	2.5 ± 2.4
Plant protein, % of total energy	9.2 ± 2.5	11.1 ± 2.4	9.0 ± 2.2	7.6 ± 2.1	6.0 ± 1.0	8.9 ± 0.4	12.8 ± 1.9
Animal to plant protein ratio	0.6 ± 0.6	0.1 ± 0.1	0.5 ± 0.1	1.4 ± 0.6	1.3 ± 0.7	0.5 ± 0.3	0.2 ± 0.2
Fat, % of total energy	18.0 ± 9.2	9.0 ± 6.4	19.2 ± 7.0	24.7 ± 8.7	25.9 ± 8.7	17.3 ± 7.6	12.4 ± 7.9
SFA, % of total energy	4.9 ± 3.0	1.9 ± 1.7	5.2 ± 2.2	7.3 ± 3.0	7.7 ± 3.0	4.7 ± 2.4	2.9 ± 2.2
MUFA, % of total energy	7.1 ± 4.5	2.6 ± 2.6	7.8 ± 3.6	10.1 ± 4.5	11.2 ± 4.5	6.8 ± 3.6	3.8 ± 3.4
PUFA, % of total energy	2.3 ± 1.6	1.6 ± 1.6	2.3 ± 1.4	2.9 ± 1.5	2.5 ± 1.2	2.1 ± 1.4	2.7 ± 2.2
Cholesterol, % of total energy	0.1 ± 0.1	0 ± 0	0.1 ± 0.1	0.2 ± 0.1	0.2 ± 0.1	0.1 ± 0.1	0.1 ± 0.1
Carbohydrates, % of total energy	67.6 ± 10.5	78.6 ± 7.0	67.1 ± 7.1	57.6 ± 9.0	59.7 ± 9.8	68.5 ± 9.2	72.2 ± 10.2
Fiber, g/day	10.9 ± 5.3	12.2 ± 5.2	10.4 ± 4.9	10.3 ± 5.4	9.6 ± 5.4	10.6 ± 5.2	12.8 ± 5.1
CMRF concentrations							
TG, mmol/L	1.6 ± 1.2	1.5 ± 1.1	1.6 ± 1.3	1.7 ± 1.3	1.6 ± 1.3	1.6 ± 1.2	1.6 ± 1.2
TC, mmol/L	4.9 ± 1.0	4.7 ± 1.0	4.9 ± 1.0	5.0 ± 1.0	4.9 ± 1.0	4.8 ± 1.0	4.8 ± 1.0
HDL-C, mmol/L	1.4 ± 0.4	1.4 ± 0.3	1.4 ± 0.3	1.4 ± 0.4	1.4 ± 0.3	1.4 ± 0.4	1.4 ± 0.4
LDL-C, mmol/L	3.0 ± 1.0	2.9 ± 1.1	3.0 ± 0.9	3.0 ± 1.0	3.0 ± 1.0	3.0 ± 1.0	2.9 ± 1.1
Non-HDL-C, mmol/L	3.5 ± 1.0	3.3 ± 1.0	3.5 ± 1.0	3.6 ± 1.0	3.5 ± 1.0	3.5 ± 1.0	3.4 ± 1.0
TC:HDL-C	3.6 ± 1.2	3.5 ± 1.1	3.6 ± 1.1	3.7 ± 1.2	3.6 ± 1.1	3.6 ± 1.2	3.6 ± 1.2
LDL-C:HDL-C	2.2 ± 0.8	2.2 ± 0.9	2.2 ± 0.8	2.3 ± 0.9	2.2 ± 0.8	2.2 ± 0.9	2.2 ± 0.9
Lipoprotein (a) (mg/L)	133.0 ± 149.2	141.3 ± 149.7	132.2 ± 152.1	129.0 ± 150.7	133.9 ± 155.7	132.6 ± 154.1	137.0 ± 148.3
Glucose (mmol/L)	5.3 ± 1.2	5.2 ± 1.3	5.3 ± 1.2	5.3 ± 1.2	5.3 ± 1.2	5.3 ± 1.2	5.3 ± 1.3
Insulin (μIU/mL)	13.8 ± 19.3	13.8 ± 21.5	13.3 ± 12.6	14.4 ± 19.1	13.7 ± 13.6	14.1 ± 21.9	14.1 ± 21.3
HOMA-IR	3.5 ± 6.0	3.5 ± 7.2	3.4 ± 4.4	3.6 ± 5.6	3.5 ± 5.2	3.6 ± 7.5	3.5 ± 5.6
HbA1c (%)	5.6 ± 0.8	5.7 ± 0.8	5.5 ± 0.8	5.5 ± 0.7	5.5 ± 0.8	5.6 ± 0.8	5.7 ± 0.8
hsCRP (mg/L)	2.5 ± 6.8	2.4 ± 5.4	2.7 ± 8.3	2.1 ± 3.8	2.3 ± 5.5	2.5 ± 6.9	2.3 ± 5.0
Uric acid (μmol/L)	306.4 ± 98.1	286.9 ± 90.7	303.4 ± 97.9	325.5 ± 103.4	315.3 ± 99.4	307.3 ± 99.6	297.3 ± 96.9

^1^ All dietary nutrients were energy adjusted. Data were presented as mean ± SD or *n* (%). CMRF, cardiometabolic risk factor; DBP, diastolic blood pressure; HDL-C, high-density lipoprotein cholesterol; HOMA-IR, homeostasis model assessment of insulin resistance; HbA1c, hemoglobin A1c; hsCRP, high-sensitive C-reactive protein; LDL-C, low-density lipoprotein cholesterol; MUFA, monounsaturated fatty acids; PUFA, polyunsaturated fatty acids; Q, quintile; SBP, systolic blood pressure; SFA, saturated fatty acids; TC, total cholesterol; TG, triglycerides.

**Table 2:** Major food sources of total, animal, and plant protein among 7886 Chinese adults who participated in the China Health and Nutrition Survey 2009.

Nutrients	Food Sources	Percentage of Contribution to Total Intake (%)
Total protein	Grains	45.7
	Red meat	15.7
	Legumes	9.2
	Fruits and vegetables	8.2
	Egg	5.6
	Fish and seafood	5.5
Animal protein	Red meat	48.8
	Egg	24.7
	Fish and seafood	15.5
	White meat	7.7
	Offal	1.8
	Dairy	1.5
Plant protein	Grains	68.9
	Legumes	13.8
	Fruits and vegetables	13.1
	Tubers	2.0
	Nuts	1.4
	Fungi and algae	0.7

**Table 3:** Associations between energy-adjusted total, animal and plant protein intakes and CMRFs in 7886 Chinese adults who participated in the China Health and Nutrition Survey 2009. ^1^

CMRF	Total Protein		Animal Protein		Plant Protein	
	β (95% CI)	*p*	β (95% CI)	*p*	β (95% CI)	*p*
Lipid and lipoprotein profiles						
TG (mmol/L)						
Model 1	0.08 (0.03, 0.12)	<0.01	0.07 (0.03, 0.11)	<0.01	−0.01 (−0.07, 0.04)	0.63
Model 2	0 (−0.06, 0.06)	0.96	0.05 (−0.01, 0.12)	0.11	−0.02 (−0.10, 0.05)	0.58
TC (mmol/L)						
Model 1	0.11 (0.07, 0.14)	<0.01	0.12 (0.09, 0.16)	<0.01	−0.09 (−0.13, −0.04)	<0.01
Model 2	0.05 (0, 0.10)	0.03	0.09 (0.04, 0.15)	<0.01	−0.04 (−0.10, 0.02)	0.22
HDL-C (mmol/L)						
Model 1	0 (−0.01, 0.01)	0.79	0 (−0.01, 0.01)	0.85	0 (−0.01, 0.02)	0.92
Model 2	0.02 (0, 0.04)	0.04	0 (−0.02, 0.02)	0.72	0.02 (0, 0.05)	0.03
LDL-C (mmol/L)						
Model 1	0.06 (0.02, 0.09)	<0.01	0.07 (0.04, 0.10)	<0.01	−0.07 (−0.11, −0.03)	<0.01
Model 2	0.03 (−0.02, 0.08)	0.26	0.05 (0, 0.11)	0.04	−0.06 (−0.12, 0)	0.07
Non-HDL-C (mmol/L)						
Model 1	0.12 (0.08, 0.16)	<0.01	0.13 (0.10, 0.17)	<0.01	−0.08 (−0.13, −0.04)	<0.01
Model 2	0.03 (−0.02, 0.08)	0.31	0.08 (0.02, 0.13)	<0.01	−0.06 (−0.13, 0)	<0.05
TC:HDL-C						
Model 1	0.11 (0.07, 0.15)	<0.01	0.10 (0.06, 0.14)	<0.01	−0.05 (−0.10, 0.01)	0.08
Model 2	0.01 (−0.05, 0.06)	0.82	0.05 (−0.01, 0.12)	0.08	−0.05 (−0.12, 0.02)	0.17
LDL-C:HDL-C						
Model 1	0.05 (0.02, 0.08)	<0.01	0.06 (0.03, 0.09)	<0.01	−0.05 (−0.09, −0.01)	<0.01
Model 2	0 (−0.04, 0.04)	0.90	0.04 (−0.01, 0.08)	0.12	−0.06 (−0.12, −0.01)	0.01
Lipoprotein (a) (mg/L)						
Model 1	−2.85 (−8.50, 2.79)	0.32	−4.67 (−9.74, 0.41)	0.07	5.94 (−0.70, 12.58)	0.08
Model 2	2.59 (−5.28, 10.45)	0.52	5.82 (−2.67, 14.31)	0.18	−0.86 (−10.60, 8.89)	0.86
Glucose homeostasis biomarkers						
Glucose (mmol/L)						
Model 1	0.02 (−0.03, 0.06)	0.44	0.04 (0, 0.08)	0.04	−0.08 (−0.13, −0.02)	<0.01
Model 2	−0.02 (−0.08, 0.04)	0.55	−0.05 (−0.12, 0.02)	0.13	−0.02 (−0.10, 0.06)	0.58
Insulin (μIU/mL)						
Model 1	0.66 (−0.06, 1.37)	0.07	0.33 (−0.32, 0.97)	0.32	0.09 (−0.76, 0.93)	0.84
Model 2	0.25 (−0.74, 1.24)	0.62	−0.17 (−1.24, 0.91)	0.76	0.38 (−0.85, 1.62)	0.54
HOMA-IR						
Model 1	0.12 (−0.10, 0.35)	0.28	0.06 (−0.14, 0.26)	0.57	−0.06 (−0.32, 0.20)	0.65
Model 2	−0.03 (−0.34, 0.28)	0.83	−0.19 (−0.53, 0.14)	0.26	−0.05 (−0.43, 0.33)	0.80
HbA1c (%)						
Model 1	0.02 (−0.01, 0.05)	0.14	−0.07 (−0.10, −0.05)	<0.01	0.13 (0.09, 0.16)	<0.01
Model 2	0.03 (−0.01, 0.07)	0.18	−0.03 (−0.08, 0.01)	0.13	0.07 (0.02, 0.12)	<0.01
hsCRP (mg/L)						
Model 1	−0.13 (−0.38, 0.13)	0.33	−0.09 (−0.31, 0.14)	0.45	0.02 (−0.28, 0.32)	0.89
Model 2	−0.20 (−0.55, 0.15)	0.25	−0.26 (−0.64, 0.11)	0.17	−0.07 (−0.50, 0.37)	0.77
Uric acid (μmol/L)						
Model 1	16.45 (12.81, 20.09)	<0.01	20.80 (17.54, 24.07)	<0.01	−12.26 (−16.56, −7.96)	<0.01
Model 2	7.44 (3.11, 11.78)	<0.01	15.32 (10.63, 20.01)	<0.01	−5.25 (−10.60, 0.11)	0.05

^1^ Data were presented as β coefficients (95% CI) per 5% energy from protein intake. Model 1 was a simple linear regression model. Model 2 was a multiple linear regression model and adjusted for potential confounders, including age, sex, BMI, urban index, region, education level, alcohol intake, smoking status, physical activity, blood pressure, cholesterol, fiber, saturated fatty acids, monounsaturated fatty acids, polyunsaturated fatty acids, other fatty acids and total energy. Model 2 was also constructed as an isocaloric substitution model, in which 5% energy from total, animal or plant protein was substituted for carbohydrates. In addition to all of the above variables, the animal protein model was also adjusted for plant protein and vice versa. CMRF, cardiometabolic risk factor; HbA1c, hemoglobin A1c; HDL-C, high-density lipoprotein cholesterol; HOMA-IR, homeostasis model assessment of insulin resistance; hsCRP, high-sensitive C-reactive protein; LDL-C, low-density lipoprotein cholesterol; TC, total cholesterol; TG, triglycerides.

**Table 4:** Associations between energy-adjusted total, animal, and plant protein intakes and CMRFs according to BMI among 7886 Chinese adults who participated in the China Health and Nutrition Survey 2009. ^1^

CMRF	Total protein		Animal protein		Plant protein	
	β (95% CI)	*p*	β (95% CI)	*p*	β (95% CI)	*p*
Lipid and lipoprotein profiles						
TG (mmol/L)						
BMI < 24 kg/m^2^	0.04 (−0.03, 0.11)	0.23	0.08 (0.01, 0.16)	0.02	−0.02 (−0.10, 0.07)	0.70
BMI ≥ 24 kg/m^2^	−0.06 (−0.18, 0.06)	0.29	0.01 (−0.13, 0.14)	0.94	−0.03 (−0.17, 0.11)	0.67
TC (mmol/L)						
BMI < 24 kg/m^2^	0.08 (0.02, 0.14)	0.01	0.12 (0.05, 0.18)	<0.01	−0.03 (−0.11, 0.05)	0.46
BMI ≥ 24 kg/m^2^	0.03 (−0.06, 0.11)	0.54	0.05 (−0.04, 0.14)	0.27	−0.03 (−0.13, 0.07)	0.51
HDL-C (mmol/L)						
BMI < 24 kg/m^2^	0.02 (0, 0.05)	0.04	0 (−0.02, 0.03)	0.77	0.04 (0.01, 0.07)	0.01
BMI ≥ 24 kg/m^2^	0.01 (−0.02, 0.03)	0.62	0 (−0.03, 0.03)	0.85	0 (−0.03, 0.04)	0.84
LDL-C (mmol/L)						
BMI < 24 kg/m^2^	0.04 (−0.02, 0.10)	0.22	0.08 (0.01, 0.14)	0.02	−0.07 (−0.15, 0.01)	0.07
BMI ≥ 24 kg/m^2^	0.02 (−0.06, 0.10)	0.62	0.02 (−0.07, 0.11)	0.68	−0.02 (−0.11, 0.08)	0.73
Non-HDL-C (mmol/L)						
BMI < 24 kg/m^2^	0.04 (−0.02, 0.11)	0.18	0.10 (0.03, 0.17)	<0.01	−0.07 (−0.15, 0.02)	0.11
BMI ≥ 24 kg/m^2^	0.01 (−0.07, 0.10)	0.78	0.04 (−0.05, 0.14)	0.36	−0.05 (−0.15, 0.05)	0.36
TC:HDL-C						
BMI < 24 kg/m^2^	0 (−0.06, 0.07)	0.93	0.07 (0, 0.14)	0.04	−0.10 (−0.18, −0.01)	0.02
BMI ≥ 24 kg/m^2^	0.02 (−0.08, 0.13)	0.67	0.03 (−0.09, 0.14)	0.65	0.03 (−0.09, 0.15)	0.65
LDL-C:HDL-C						
BMI < 24 kg/m^2^	−0.01 (−0.06, 0.04)	0.76	0.05 (−0.01, 0.11)	0.08	−0.11 (−0.17, −0.04)	<0.01
BMI ≥ 24 kg/m^2^	0.02 (−0.05, 0.09)	0.64	0.01 (−0.06, 0.09)	0.73	0.01 (−0.08, 0.09)	0.84
Lipoprotein (a) (mg/L)						
BMI < 24 kg/m^2^	2.38 (−8.04, 12.81)	0.65	7.01 (−4.17, 18.19)	0.22	2.19 (−11.09, 15.46)	0.75
BMI ≥ 24 kg/m^2^	2.22 (−9.72, 14.16)	0.72	3.85 (−9.18, 16.88)	0.56	−5.38 (−19.62, 8.85)	0.46
Glucose homeostasis biomarkers						
Glucose (mmol/L)						
BMI < 24 kg/m^2^	0 (−0.07, 0.07)	1.00	−0.02 (−0.09, 0.05)	0.59	−0.03 (−0.12, 0.06)	0.46
BMI ≥ 24 kg/m^2^	−0.05 (−0.16, 0.07)	0.44	−0.10 (−0.23, 0.03)	0.12	0 (−0.13, 0.14)	0.95
Insulin (μIU/mL)						
BMI < 24 kg/m^2^	0.34 (−1.05, 1.73)	0.63	−0.22 (−1.71, 1.28)	0.78	0.40 (−1.37, 2.17)	0.66
BMI ≥ 24 kg/m^2^	0.24 (−1.10, 1.59)	0.72	−0.07 (−1.54, 1.41)	0.93	0.59 (−1.01, 2.18)	0.47
HOMA-IR						
BMI < 24 kg/m^2^	0.03 (−0.38, 0.43)	0.90	−0.14 (−0.57, 0.29)	0.52	−0.10 (−0.61, 0.41)	0.69
BMI ≥ 24 kg/m^2^	−0.12 (−0.61, 0.37)	0.64	−0.30 (−0.84, 0.23)	0.27	0.07 (−0.51, 0.66)	0.81
HbA1c (%)						
BMI < 24 kg/m^2^	0.03 (−0.02, 0.08)	0.19	−0.02 (−0.07, 0.03)	0.49	0.06 (0, 0.13)	<0.05
BMI ≥ 24 kg/m^2^	0.02 (−0.05, 0.09)	0.58	−0.06 (−0.13, 0.02)	0.14	0.08 (0, 0.16)	<0.05
hsCRP (mg/L)						
BMI < 24 kg/m^2^	−0.28 (−0.77, 0.21)	0.27	−0.38 (−0.90, 0.15)	0.16	0.07 (−0.56, 0.70)	0.82
BMI ≥ 24 kg/m^2^	−0.07 (−0.53, 0.38)	0.75	−0.05 (−0.55, 0.45)	0.85	−0.26 (−0.80, 0.29)	0.36
Uric acid (μmol/L)						
BMI < 24 kg/m^2^	8.35 (3.16, 13.54)	<0.01	15.82 (10.27, 21.38)	<0.01	−5.86 (−12.45, 0.74)	0.08
BMI ≥ 24 kg/m^2^	6.82 (−0.87, 14.51)	0.08	14.91 (6.46, 23.36)	<0.01	−3.82 (−12.93, 5.30)	0.41

^1^ Data were presented as β coefficients (95% CI) per 5% energy from protein intake. The model was a multiple linear regression model, and adjusted for potential confounders, including age, sex, urban index, region, education level, alcohol intake, smoking status, physical activity, blood pressure, cholesterol, fiber, saturated fatty acids, monounsaturated fatty acids, polyunsaturated fatty acids, other fatty acids and total energy. The model was also constructed as an isocaloric substitution model, in which 5% energy from total, animal or plant protein was substituted for carbohydrates. In addition to all of the above variables, the animal protein model was also adjusted for plant protein and vice versa. CMRF, cardiometabolic risk factor; HbA1c, hemoglobin A1c; HDL-C, high-density lipoprotein cholesterol; HOMA-IR, homeostasis model assessment of insulin resistance; hsCRP, high-sensitive C-reactive protein; LDL-C, low-density lipoprotein cholesterol; TC, total cholesterol; TG, triglycerides.

**Table 5:** Associations between energy-adjusted total, animal and plant protein intakes and CMRFs according to sex among 7886 Chinese adults who participated in the China Health and Nutrition Survey 2009. ^1^

CMRF	Total Protein		Animal Protein		Plant Protein	
	β (95% CI)	*p*	β (95% CI)	*p*	β (95% CI)	*p*
Lipid and lipoprotein profiles						
TG (mmol/L)						
Women	0.02 (−0.06, 0.10)	0.60	0.06 (−0.03, 0.14)	0.17	−0.01 (−0.10, 0.08)	0.87
Men	−0.04 (−0.14, 0.06)	0.45	0.03 (−0.07, 0.14)	0.53	−0.04 (−0.16, 0.09)	0.55
TC (mmol/L)						
Women	0.06 (0, 0.13)	0.07	0.08 (0, 0.15)	0.04	0 (−0.08, 0.08)	0.95
Men	0.03 (−0.04, 0.10)	0.37	0.09 (0.02, 0.17)	0.02	−0.09 (−0.18, 0)	<0.05
HDL-C (mmol/L)						
Women	0.01 (−0.01, 0.03)	0.43	−0.01 (−0.04, 0.02)	0.41	0.01 (−0.02, 0.04)	0.56
Men	0.03 (0, 0.05)	0.03	0.02 (−0.01, 0.05)	0.18	0.04 (0.01, 0.08)	0.01
LDL-C (mmol/L)						
Women	0.06 (−0.01, 0.12)	0.10	0.05 (−0.02, 0.13)	0.16	0.03 (−0.05, 0.11)	0.52
Men	−0.01 (−0.08, 0.06)	0.78	0.04 (−0.03, 0.12)	0.25	−0.17 (−0.26, −0.07)	<0.01
Non-HDL-C (mmol/L)						
Women	0.04 (−0.03, 0.11)	0.26	0.07 (−0.01, 0.15)	0.08	0 (−0.09, 0.08)	0.97
Men	0 (−0.07, 0.08)	0.93	0.07 (−0.01, 0.14)	0.09	−0.14 (−0.23, −0.04)	<0.01
TC:HDL-C						
Women	0.03 (−0.04, 0.10)	0.44	0.08 (0, 0.16)	0.05	0 (−0.09, 0.08)	0.96
Men	−0.03 (−0.12, 0.06)	0.50	0.02 (−0.08, 0.11)	0.74	−0.11 (−0.22, 0)	<0.05
LDL-C:HDL-C						
Women	0.03 (−0.02, 0.09)	0.23	0.05 (−0.01, 0.12)	0.08	0.02 (−0.04, 0.09)	0.47
Men	−0.05 (−0.11, 0.01)	0.13	0.01 (−0.06, 0.07)	0.84	−0.18 (−0.26, −0.10)	<0.01
Lipoprotein (a) (mg/L)						
Women	−1.05 (−12.32, 10.21)	0.85	3.49 (−9.00, 15.98)	0.58	−2.26 (−15.92, 11.39)	0.75
Men	6.22 (−4.73, 17.18)	0.27	7.52 (−4.01, 19.06)	0.20	1.06 (−12.86, 14.98)	0.88
Glucose homeostasis biomarkers						
Glucose (mmol/L)						
Women	−0.04 (−0.12, 0.04)	0.30	−0.05 (−0.13, 0.04)	0.27	−0.04 (−0.13, 0.06)	0.45
Men	0 (−0.10, 0.10)	0.94	−0.05 (−0.15, 0.06)	0.37	−0.01 (−0.13, 0.12)	0.91
Insulin (μIU/mL)						
Women	0.84 (−0.49, 2.16)	0.22	0.63 (−0.85, 2.11)	0.40	0.18 (−1.43, 1.79)	0.83
Men	−0.49 (−2.00, 1.01)	0.52	−0.96 (−2.54, 0.63)	0.24	0.48 (−1.43, 2.39)	0.62
HOMA-IR						
Women	0.10 (−0.34, 0.55)	0.66	−0.01 (−0.51, 0.48)	0.96	−0.10 (−0.64, 0.44)	0.73
Men	−0.21 (−0.64, 0.22)	0.34	−0.37 (−0.83, 0.08)	0.10	−0.03 (−0.58, 0.51)	0.91
HbA1c (%)						
Women	0.02 (−0.03, 0.08)	0.44	−0.04 (−0.10, 0.03)	0.26	0.06 (0, 0.13)	0.06
Men	0.03 (−0.02, 0.09)	0.25	−0.03 (−0.08, 0.03)	0.41	0.08 (0.01, 0.15)	0.03
hsCRP (mg/L)						
Women	−0.23 (−0.64, 0.18)	0.26	−0.11 (−0.57, 0.35)	0.64	−0.18 (−0.68, 0.32)	0.48
Men	−0.16 (−0.74, 0.42)	0.59	−0.38 (−1.00, 0.23)	0.22	0.07 (−0.67, 0.81)	0.85
Uric acid (μmol/L)						
Women	7.25 (2.09, 12.42)	<0.01	14.81 (9.05, 20.56)	<0.01	−4.01 (−10.23, 2.22)	0.21
Men	6.76 (−0.30, 13.83)	0.06	14.65 (7.22, 22.07)	<0.01	−6.79 (−15.75, 2.18)	0.14

^1^ Data were presented as β coefficients (95% CI) per 5% energy from protein intake. The model was a multiple linear regression model, and adjusted for potential confounders, including age, BMI, urban index, region, education level, alcohol intake, smoking status, physical activity, blood pressure, cholesterol, fiber, saturated fatty acids, monounsaturated fatty acids, polyunsaturated fatty acids, other fatty acids and total energy. The model was also constructed as an isocaloric substitution model, in which 5% energy from total, animal or plant protein was substituted for carbohydrates. In addition to all of the above variables, the animal protein model was also adjusted for plant protein and vice versa. CMRF, cardiometabolic risk factor; HbA1c, hemoglobin A1c; HDL-C, high-density lipoprotein cholesterol; HOMA-IR, homeostasis model assessment of insulin resistance; hsCRP, high-sensitive C-reactive protein; LDL-C, low-density lipoprotein cholesterol; TC, total cholesterol; TG, triglycerides.

**Table 6:** Associations between energy-adjusted total, animal and plant protein intakes and CMRFs according to age among 7886 Chinese adults who participated in the China Health and Nutrition Survey 2009. ^1^

CMRF	Total Protein		Animal Protein		Plant Protein	
	β (95% CI)	*p*	β (95% CI)	*p*	β (95% CI)	*p*
Lipid and lipoprotein profiles						
TG (mmol/L)						
Age < 60 years	0.01 (−0.07, 0.08)	0.85	0.08 (0, 0.16)	0.05	0.01 (−0.08, 0.10)	0.86
Age ≥ 60 years	−0.01 (−0.12, 0.10)	0.86	−0.01 (−0.13, 0.11)	0.90	−0.07 (−0.20, 0.06)	0.28
TC (mmol/L)						
Age < 60 years	0.05 (−0.01, 0.10)	0.12	0.09 (0.03, 0.15)	<0.01	−0.02 (−0.09, 0.06)	0.65
Age ≥ 60 years	0.07 (−0.03, 0.17)	0.15	0.10 (−0.01, 0.21)	0.09	−0.07 (−0.19, 0.04)	0.21
HDL-C (mmol/L)						
Age < 60 years	0.01 (−0.01, 0.03)	0.27	0 (−0.03, 0.02)	0.72	0.02 (−0.01, 0.04)	0.13
Age ≥ 60 years	0.03 (0, 0.07)	0.08	0.02 (−0.02, 0.06)	0.35	0.03 (−0.01, 0.07)	0.15
LDL-C (mmol/L)						
Age < 60 years	0.01 (−0.04, 0.07)	0.61	0.05 (−0.01, 0.11)	0.14	−0.06 (−0.13, 0.01)	0.08
Age ≥ 60 years	0.06 (−0.05, 0.16)	0.30	0.06 (−0.05, 0.18)	0.28	−0.03 (−0.16, 0.09)	0.58
Non-HDL-C (mmol/L)						
Age < 60 years	0.02 (−0.04, 0.08)	0.42	0.08 (0.02, 0.15)	0.01	−0.04 (−0.12, 0.03)	0.29
Age ≥ 60 years	0.04 (−0.06, 0.14)	0.45	0.06 (−0.05, 0.18)	0.27	−0.09 (−0.21, 0.04)	0.17
TC:HDL-C						
Age < 60 years	0.02 (−0.04, 0.09)	0.51	0.08 (0.01, 0.15)	0.04	−0.01 (−0.10, 0.07)	0.75
Age ≥ 60 years	−0.03 (−0.13, 0.08)	0.64	0 (−0.12, 0.12)	0.98	−0.11 (−0.24, 0.01)	0.08
LDL-C:HDL-C						
Age < 60 years	−0.01 (−0.05, 0.04)	0.81	0.04 (−0.01, 0.09)	0.12	−0.07 (−0.13, −0.01)	0.03
Age ≥ 60 years	0.01 (−0.08, 0.10)	0.90	0.02 (−0.08, 0.12)	0.71	−0.05 (−0.15, 0.06)	0.36
Lipoprotein (a) (mg/L)						
Age < 60 years	1.59 (−7.41, 10.58)	0.73	4.72 (−4.88, 14.33)	0.34	0.05 (−11.30, 11.40)	0.99
Age ≥ 60 years	5.40 (−10.92, 21.72)	0.52	9.87 (−8.46, 28.20)	0.29	−2.98 (−22.24, 16.29)	0.76
Glucose homeostasis biomarkers						
Glucose (mmol/L)						
Age < 60 years	−0.02 (−0.09, 0.05)	0.60	−0.04 (−0.11, 0.04)	0.34	0.03 (−0.05, 0.12)	0.47
Age ≥ 60 years	−0.02 (−0.16, 0.11)	0.74	−0.10 (−0.26, 0.05)	0.18	−0.13 (−0.29, 0.03)	0.10
Insulin (*μ*IU/mL)						
Age < 60 years	0.18 (−0.80, 1.17)	0.71	−0.12 (−1.17, 0.94)	0.83	0.84 (−0.41, 2.08)	0.19
Age ≥ 60 years	0.91 (−1.75, 3.57)	0.50	0.42 (−2.56, 3.39)	0.78	−0.58 (−3.69, 2.54)	0.72
HOMA-IR						
Age < 60 years	−0.04 (−0.33, 0.26)	0.81	−0.16 (−0.48, 0.16)	0.32	0.13 (−0.25, 0.51)	0.50
Age ≥ 60 years	0.10 (−0.75, 0.96)	0.82	−0.08 (−1.04, 0.87)	0.87	−0.45 (−1.45, 0.55)	0.37
HbA1c (%)						
Age < 60 years	0.05 (0, 0.09)	<0.05	0 (−0.05, 0.05)	0.97	0.09 (0.03, 0.15)	<0.01
Age ≥ 60 years	−0.05 (−0.13, 0.04)	0.26	−0.17 (−0.27, −0.08)	<0.01	0.03 (−0.07, 0.13)	0.57
hsCRP (mg/L)						
Age < 60 years	−0.12 (−0.48, 0.24)	0.50	−0.27 (−0.65, 0.12)	0.18	0.27 (−0.19, 0.72)	0.25
Age ≥ 60 years	−0.46 (−1.34, 0.42)	0.30	−0.26 (−1.24, 0.72)	0.60	−0.86 (−1.89, 0.17)	0.10
Uric acid (μmol/L)						
Age < 60 years	7.62 (2.64, 12.61)	<0.01	15.55 (10.21, 20.89)	<0.01	−4.81 (−11.09, 1.48)	0.13
Age ≥ 60 years	7.76 (−0.91, 16.43)	0.08	15.21 (5.54, 24.88)	<0.01	−5.43 (−15.57, 4.72)	0.29

^1^ Data were presented as β coefficients (95% CI) per 5% energy from protein intake. The model was a multiple linear regression model, and adjusted for potential confounders, including sex, BMI, urban index, region, education level, alcohol intake, smoking status, physical activity, blood pressure, cholesterol, fiber, saturated fatty acids, monounsaturated fatty acids, polyunsaturated fatty acids, other fatty acids and total energy. The model was also constructed as an isocaloric substitution model, in which 5% energy from total, animal or plant protein was substituted for carbohydrate. In addition to all of the above variables, the animal protein model was also adjusted for plant protein and vice versa. CMRF, cardiometabolic risk factor; HbA1c, hemoglobin A1c; HDL-C, high-density lipoprotein cholesterol; HOMA-IR, homeostasis model assessment of insulin resistance; hsCRP, high-sensitive C-reactive protein; LDL-C, low-density lipoprotein cholesterol; TC, total cholesterol; TG, triglycerides.

**Table 7:** Associations between energy-adjusted total, animal and plant protein intakes and CMRFs according to region among 7886 Chinese adults who participated in the China Health and Nutrition Survey 2009. ^1^

CMRF	Total Protein		Animal Protein		Plant Protein	
	β (95% CI)	*p*	β (95% CI)	*p*	β (95% CI)	*p*
Lipid and lipoprotein profiles						
TG (mmol/L)						
Northern	0.02 (−0.09, 0.14)	0.71	0.09 (−0.03, 0.22)	0.14	−0.03 (−0.15, 0.09)	0.59
Southern	0.01 (−0.06, 0.09)	0.75	0.04 (−0.04, 0.12)	0.35	0.04 (−0.07, 0.14)	0.52
TC (mmol/L)						
Northern	0.12 (0.04, 0.21)	<0.01	0.14 (0.05, 0.24)	<0.01	−0.01 (−0.10, 0.08)	0.87
Southern	0.05 (−0.01, 0.12)	0.08	0.10 (0.03, 0.17)	<0.01	0.01 (−0.08, 0.09)	0.90
HDL-C (mmol/L)						
Northern	0.02 (−0.01, 0.05)	0.15	0.02 (−0.01, 0.05)	0.26	0.01 (−0.02, 0.04)	0.63
Southern	0.01 (−0.01, 0.03)	0.35	0.00 (−0.03, 0.02)	0.90	0.04 (0.01, 0.07)	0.01
LDL-C (mmol/L)						
Northern	0.09 (−0.01, 0.18)	0.06	0.06 (−0.03, 0.16)	0.20	0.00 (−0.09, 0.10)	0.95
Southern	0.02 (−0.04, 0.08)	0.48	0.07 (0, 0.13)	0.04	−0.07 (−0.15, 0.01)	0.11
Non-HDL-C (mmol/L)						
Northern	0.08 (−0.01, 0.18)	0.08	0.11 (0.01, 0.21)	0.04	−0.02 (−0.11, 0.08)	0.75
Southern	0.04 (−0.02, 0.11)	0.19	0.10 (0.03, 0.17)	<0.01	−0.03 (−0.12, 0.06)	0.46
TC:HDL-C						
Northern	0.06 (−0.05, 0.17)	0.28	0.07 (−0.05, 0.19)	0.27	0.01 (−0.10, 0.12)	0.84
Southern	0.02 (−0.05, 0.09)	0.57	0.06 (−0.01, 0.14)	0.10	−0.05 (−0.14, 0.05)	0.36
LDL-C:HDL-C						
Northern	0.04 (−0.04, 0.12)	0.30	0.02 (−0.06, 0.11)	0.60	0.02 (−0.07, 0.10)	0.71
Southern	0 (−0.05, 0.05)	0.91	0.05 (−0.01, 0.10)	0.09	−0.10 (−0.18, −0.03)	<0.01
Lipoprotein (a) (mg/L)						
Northern	1.50 (−12.03, 15.04)	0.83	0.69 (−14.15, 15.53)	0.93	−3.06 (−17.06, 10.93)	0.67
Southern	2.69 (−7.21, 12.60)	0.59	8.26 (−2.45, 18.97)	0.13	−2.05 (−15.96, 11.86)	0.77
Glucose homeostasis biomarkers						
Glucose (mmol/L)						
Northern	−0.08 (−0.20, 0.03)	0.17	−0.18 (−0.31, −0.05)	<0.01	−0.06 (−0.19, 0.06)	0.30
Southern	0.03 (−0.05, 0.10)	0.47	0.02 (−0.06, 0.09)	0.71	0.06 (−0.04, 0.16)	0.25
Insulin (μIU/mL)						
Northern	0.80 (−0.87, 2.47)	0.35	0.17 (−1.66, 1.99)	0.86	0.32 (−1.41, 2.05)	0.72
Southern	0.07 (−1.21, 1.36)	0.91	−0.42 (−1.82, 0.97)	0.55	0.95 (−0.84, 2.75)	0.30
HOMA-IR						
Northern	0.11 (−0.48, 0.70)	0.71	−0.17 (−0.81, 0.48)	0.61	−0.10 (−0.71, 0.51)	0.75
Southern	−0.07 (−0.43, 0.29)	0.71	−0.23 (−0.62, 0.16)	0.25	0.18 (−0.33, 0.68)	0.50
HbA1c (%)						
Northern	−0.05 (−0.12, 0.02)	0.19	−0.16 (−0.23, −0.08)	<0.01	−0.02 (−0.09, 0.06)	0.65
Southern	0.06 (0.02, 0.11)	<0.01	0.03 (−0.03, 0.08)	0.33	0.17 (0.10, 0.23)	<0.01
hsCRP (mg/L)						
Northern	0.04 (−0.39, 0.48)	0.84	−0.02 (−0.50, 0.46)	0.95	−0.07 (−0.52, 0.39)	0.77
Southern	−0.33 (−0.83, 0.18)	0.20	−0.40 (−0.95, 0.15)	0.15	−0.03 (−0.74, 0.68)	0.94
Uric acid (μmol/L)						
Northern	6.31 (−1.38, 14.00)	0.11	21.38 (13.01, 29.75)	<0.01	−9.23 (−17.16, −1.30)	0.02
Southern	10.78 (5.25, 16.30)	<0.01	14.08 (8.07, 20.09)	<0.01	1.82 (−5.89, 9.53)	0.64

^1^ Data were presented as β coefficients (95% CI) per 5% energy from protein intake. Northern regions included Liaoning, Heilongjiang, Shandong, and Henan provinces, and southern regions included Jiangsu, Hubei, Hunan, Guizhou, and Guangxi provinces. The model was a multiple linear regression model, and adjusted for potential confounders, including age, sex, BMI, urban index, education level, alcohol intake, smoking status, physical activity, blood pressure, cholesterol, fiber, saturated fatty acids, monounsaturated fatty acids, polyunsaturated fatty acids, other fatty acids and total energy. The model was also constructed as an isocaloric substitution model, in which 5% energy from total, animal or plant protein was substituted for carbohydrates. In addition to all of the above variables, the animal protein model was also adjusted for plant protein and vice versa. CMRF, cardiometabolic risk factor; HbA1c, hemoglobin A1c; HDL-C, high-density lipoprotein cholesterol; HOMA-IR, homeostasis model assessment of insulin resistance; hsCRP, high-sensitive C-reactive protein; LDL-C, low-density lipoprotein cholesterol; TC, total cholesterol; TG, triglycerides.

## Data Availability

All data used during the study are available online (https://www.cpc.unc.edu/projects/china).

## Supplementary Materials

The following are available online at https://www.mdpi.com/2072-6643/13/2/336/s1, Table S1: Associations between the animal to plant protein ratio and cardiometabolic risk factors in 7886 Chinese adults who participated in the China Health and Nutrition Survey 2009; Table S2: Associations between the animal to plant protein ratio and cardiometabolic risk factors according to BMI among 7886 Chinese adults who participated in the China Health and Nutrition Survey 2009; Table S3: Associations between the animal to plant protein ratio and cardiometabolic risk factors according to sex among 7886 Chinese adults who participated in the China Health and Nutrition Survey 2009; Table S4: Associations between the animal to plant protein ratio and cardiometabolic risk factors according to age among 7886 Chinese adults who participated in the China Health and Nutrition Survey 2009; Table S5: Associations between the animal to plant protein ratio and cardiometabolic risk factors according to region among 7886 Chinese adults who participated in the China Health and Nutrition Survey 2009; Figure S1: Participant flow diagram.

## Abbreviations

BCAA	branched-chain amino acid
BMI	body mass index
CHNS	China Health and Nutrition Survey
CMRF	cardiometabolic risk factor
DBP	diastolic blood pressure
EAAs	essential amino acids
HbA1c	hemoglobin A1c
HDL-C	high-density lipoprotein cholesterol
HOMA-IR	homeostasis model assessment of insulin resistance
hsCRP	high-sensitive C-reactive protein
LDL-C	low-density lipoprotein cholesterol
MUFA	monounsaturated fatty acids
NEAAs	nonessential amino acids
PUFA	polyunsaturated fatty acids
RCT	randomized, double-blind controlled trial
SBP	systolic blood pressure
SFA	saturated fatty acids
TMAO	trimethylamine-N-oxide
TC	total cholesterol
TG	triglycerides
