# Supplementary material for: Associations between Dietary Animal and Plant Protein Intake and Cardiometabolic Risk Factors—A Cross-Sectional Study in China Health and Nutrition Survey
Source: Nutrients. 2021 Jan 23;13(2):336. doi: 10.3390/nu13020336 (PMC7912240; doi:10.3390/nu13020336)

Supplemental Material

Table S1. Associations between the animal to plant protein ratio and cardiometabolic risk factors in 7886 Chinese adults who participated in China Health and Nutrition Survey 2009. [1]

Cardiometabolic risk factors	The animal to plant protein ratio	
	β (95% CI)	*p*
Lipid and lipoprotein profiles		
TG (mmol/L)		
Model 1	0.06 (0.02, 0.11)	0.01
Model 2	0.05 (-0.02, 0.12)	0.16
TC (mmol/L)		
Model 1	0.13 (0.09, 0.17)	< 0.01
Model 2	0.10 (0.04, 0.15)	< 0.01
HDL-C (mmol/L)		
Model 1	0 (-0.01, 0.02)	0.75
Model 2	0 (-0.02, 0.02)	0.99
LDL-C (mmol/L)		
Model 1	0.08 (0.04, 0.12)	< 0.01
Model 2	0.09 (0.03, 0.14)	< 0.01
Non-HDL-C (mmol/L)		
Model 1	0.13 (0.09, 0.17)	< 0.01
Model 2	0.08 (0.02, 0.14)	0.01
TC:HDL-C		
Model 1	0.09 (0.05, 0.14)	< 0.01
Model 2	0.05 (-0.01, 0.12)	0.13
LDL-C:HDL-C		
Model 1	0.06 (0.03, 0.10)	< 0.01
Model 2	0.06 (0.01, 0.11)	0.01
Lipoprotein (a) (mg/L)		
Model 1	-4.72 (-10.66, 1.23)	0.12
Model 2	6.53 (-2.53, 15.58)	0.16
Glucose homeostasis biomarkers		
Glucose (mmol/L)		
Model 1	0.04 (-0.01, 0.09)	0.09
Model 2	-0.05 (-0.13, 0.02)	0.13
Insulin (μIU/mL)		
Model 1	0.37 (-0.39, 1.12)	0.34
Model 2	-0.07 (-1.22, 1.07)	0.90
HOMA-IR		
Model 1	0.06 (-0.17, 0.30)	0.60
Model 2	-0.14 (-0.50, 0.22)	0.44
HbA1c (%)		
Model 1	-0.09 (-0.13, -0.06)	< 0.01
Model 2	-0.06 (-0.11, -0.02)	0.01
hsCRP (mg/L)		
Model 1	-0.11 (-0.37, 0.16)	0.43
Model 2	-0.23 (-0.63, 0.17)	0.26
Uric acid (μmol/L)		
Model 1	21.50 (17.68, 25.33)	< 0.01
Model 2	15.53 (10.53, 20.53)	< 0.01

[1] Data were presented as β coefficients (95% CI). Model 1 was a simple linear regression model. Model 2 was a multiple linear regression model and adjusted for potential confounders,

including age, sex, BMI, urban index, region, education level, alcohol intake, smoking status, physical activity, blood pressure, cholesterol, fiber, saturated fatty acids, monounsaturated fatty acids, polyunsaturated fatty acids, other fatty acids and total energy. CMRF, cardiometabolic risk factor; HbA1c, hemoglobin A1c; HDL-C, high-density lipoprotein cholesterol; HOMA-IR, homeostasis model assessment of insulin resistance; hsCRP, high-sensitive C-reactive protein; LDL-C, low-density lipoprotein cholesterol; TC, total cholesterol; TG, triglycerides.

Supplemental Material

Table S2. Associations between the animal to plant protein ratio and cardiometabolic risk factors according to BMI among 7886 Chinese adults who participated in China Health and Nutrition Survey 2009. [1]

Cardiometabolic risk factors	The animal to plant protein ratio	
	β (95% CI)	*p*
Lipid and lipoprotein profiles		
TG (mmol/L)		
BMI < 24 kg/m^2	0.08 (0.01, 0.16)	0.03
BMI ≥ 24 kg/m^2	-0.02 (-0.16, 0.13)	0.84
TC (mmol/L)		
BMI < 24 kg/m^2	0.12 (0.06, 0.19)	< 0.01
BMI ≥ 24 kg/m^2	0.04 (-0.06, 0.15)	0.39
HDL-C (mmol/L)		
BMI < 24 kg/m^2	-0.01 (-0.03, 0.02)	0.54
BMI ≥ 24 kg/m^2	0.02 (-0.01, 0.05)	0.27
LDL-C (mmol/L)		
BMI < 24 kg/m^2	0.11 (0.05, 0.18)	< 0.01
BMI ≥ 24 kg/m^2	0.03 (-0.07, 0.14)	0.51
Non-HDL-C (mmol/L)		
BMI < 24 kg/m^2	0.12 (0.05, 0.20)	< 0.01
BMI ≥ 24 kg/m^2	0 (-0.11, 0.10)	0.95
TC:HDL-C		
BMI < 24 kg/m^2	0.11 (0.04, 0.18)	< 0.01
BMI ≥ 24 kg/m^2	-0.06 (-0.19, 0.06)	0.32
LDL-C:HDL-C		
BMI < 24 kg/m^2	0.09 (0.03, 0.15)	< 0.01
BMI ≥ 24 kg/m^2	0 (-0.09, 0.09)	0.97
Lipoprotein (a) (mg/L)		
BMI < 24 kg/m^2	8.26 (-3.33, 19.86)	0.16
BMI ≥ 24 kg/m^2	3.35 (-11.25, 17.96)	0.65
Glucose homeostasis biomarkers		
Glucose (mmol/L)		
BMI < 24 kg/m^2	-0.01 (-0.08, 0.07)	0.90
BMI ≥ 24 kg/m^2	-0.16 (-0.30, -0.01)	0.03
Insulin (μIU/mL)		
BMI < 24 kg/m^2	-0.10 (-1.65, 1.45)	0.90
BMI ≥ 24 kg/m^2	-0.11 (-1.75, 1.54)	0.90
HOMA-IR		
BMI < 24 kg/m^2	-0.04 (-0.49, 0.40)	0.85
BMI ≥ 24 kg/m^2	-0.37 (-0.97, 0.23)	0.23
HbA1c (%)		
BMI < 24 kg/m^2	-0.04 (-0.10, 0.01)	0.13
BMI ≥ 24 kg/m^2	-0.10 (-0.18, -0.01)	0.03
hsCRP (mg/L)		
BMI < 24 kg/m^2	-0.29 (-0.84, 0.26)	0.30
BMI ≥ 24 kg/m^2	-0.08 (-0.65, 0.48)	0.77
Uric acid (μmol/L)		
BMI < 24 kg/m^2	15.76 (10.00, 21.52)	< 0.01
BMI ≥ 24 kg/m^2	14.96 (5.45, 24.46)	< 0.01

[1] Data were presented as β coefficients (95% CI). Model 1 was a simple linear regression model. Model 2 was a multiple linear regression model and adjusted for potential confounders,

including age, sex, urban index, region, education level, alcohol intake, smoking status, physical activity, blood pressure, cholesterol, fiber, saturated fatty acids, monounsaturated fatty acids, polyunsaturated fatty acids, other fatty acids and total energy. CMRF, cardiometabolic risk factor; HbA1c, hemoglobin A1c; HDL-C, high-density lipoprotein cholesterol; HOMA-IR, homeostasis model assessment of insulin resistance; hsCRP, high-sensitive C-reactive protein; LDL-C, low-density lipoprotein cholesterol; TC, total cholesterol; TG, triglycerides.

Supplemental Material

Table S3. Associations between the animal to plant protein ratio and cardiometabolic risk factors according to sex among 7886 Chinese adults who participated in China Health and Nutrition Survey 2009. [1]

Cardiometabolic risk factors	The animal to plant protein ratio	
	β (95% CI)	*p*
Lipid and lipoprotein profiles		
TG (mmol/L)		
Women	0.06 (-0.03, 0.15)	0.20
Men	0.02 (-0.09, 0.13)	0.71
TC (mmol/L)		
Women	0.08 (0, 0.16)	0.06
Men	0.10 (0.02, 0.18)	0.01
HDL-C (mmol/L)		
Women	0 (-0.03, 0.02)	0.78
Men	0 (-0.02, 0.03)	0.79
LDL-C (mmol/L)		
Women	0.05 (-0.03, 0.13)	0.26
Men	0.12 (0.04, 0.20)	< 0.01
Non-HDL-C (mmol/L)		
Women	0.06 (-0.03, 0.14)	0.19
Men	0.08 (0, 0.16)	0.06
TC:HDL-C		
Women	0.07 (-0.01, 0.16)	0.09
Men	0.01 (-0.08, 0.11)	0.77
LDL-C:HDL-C		
Women	0.04 (-0.03, 0.10)	0.26
Men	0.08 (0.01, 0.15)	0.03
Lipoprotein (a) (mg/L)		
Women	3.54 (-9.89, 16.97)	0.61
Men	8.29 (-3.91, 20.49)	0.18
Glucose homeostasis biomarkers		
Glucose (mmol/L)		
Women	-0.04 (-0.13, 0.05)	0.36
Men	-0.06 (-0.17, 0.05)	0.29
Insulin (μIU/mL)		
Women	0.58 (-1.01, 2.17)	0.47
Men	-0.66 (-2.34, 1.01)	0.44
HOMA-IR		
Women	0.01 (-0.52, 0.54)	0.97
Men	-0.28 (-0.76, 0.20)	0.25
HbA1c (%)		
Women	-0.06 (-0.13, 0)	0.06
Men	-0.05 (-0.12, 0.01)	0.10
hsCRP (mg/L)		
Women	-0.20 (-0.69, 0.29)	0.42
Men	-0.24 (-0.89, 0.40)	0.46
Uric acid (μmol/L)		
Women	15.70 (9.50, 21.90)	< 0.01
Men	14.13 (6.29, 21.96)	< 0.01

[1] Data were presented as β coefficients (95% CI). Model 1 was a simple linear regression model. Model 2 was a multiple linear regression model and adjusted for potential confounders,

including age, BMI, urban index, region, education level, alcohol intake, smoking status, physical activity, blood pressure, cholesterol, fiber, saturated fatty acids, monounsaturated fatty acids, polyunsaturated fatty acids, other fatty acids and total energy. CMRF, cardiometabolic risk factor; HbA1c, hemoglobin A1c; HDL-C, high-density lipoprotein cholesterol; HOMA-IR, homeostasis model assessment of insulin resistance; hsCRP, high-sensitive C-reactive protein; LDL-C, low-density lipoprotein cholesterol; TC, total cholesterol; TG, triglycerides.

Supplemental Material

Table S4. Associations between the animal to plant protein ratio and cardiometabolic risk factors according to age among 7886 Chinese adults who participated in China Health and Nutrition Survey 2009. [1]

Cardiometabolic risk factors	The animal to plant protein ratio	
	β (95% CI)	*p*
Lipid and lipoprotein profiles		
TG (mmol/L)		
Age < 60 years	0.05 (-0.03, 0.14)	0.20
Age ≥ 60 years	0.05 (-0.09, 0.18)	0.50
TC (mmol/L)		
Age < 60 years	0.06 (0, 0.13)	0.06
Age ≥ 60 years	0.17 (0.05, 0.30)	0.01
HDL-C (mmol/L)		
Age < 60 years	-0.01 (-0.03, 0.01)	0.34
Age ≥ 60 years	0.03 (-0.03, 0.08)	0.22
LDL-C (mmol/L)		
Age < 60 years	0.06 (0, 0.13)	0.05
Age ≥ 60 years	0.12 (-0.01, 0.25)	0.07
Non-HDL-C (mmol/L)		
Age < 60 years	0.06 (0, 0.13)	0.07
Age ≥ 60 years	0.11 (-0.01, 0.24)	0.08
TC:HDL-C		
Age < 60 years	0.06 (-0.02, 0.13)	0.14
Age ≥ 60 years	0.04 (-0.10, 0.17)	0.61
LDL-C:HDL-C		
Age < 60 years	0.07 (0.01, 0.12)	0.01
Age ≥ 60 years	0.03 (-0.08, 0.14)	0.64
Lipoprotein (a) (mg/L)		
Age < 60 years	3.24 (-6.88, 13.36)	0.53
Age ≥ 60 years	15.20 (-5.25, 35.66)	0.15
Glucose homeostasis biomarkers		
Glucose (mmol/L)		
Age < 60 years	-0.07 (-0.15, 0.01)	0.07
Age ≥ 60 years	-0.03 (-0.20, 0.14)	0.76
Insulin (μIU/mL)		
Age < 60 years	0.01 (-1.10, 1.13)	0.98
Age ≥ 60 years	0.06 (-3.26, 3.38)	0.97
HOMA-IR		
Age < 60 years	-0.14 (-0.48, 0.19)	0.40
Age ≥ 60 years	-0.03 (-1.10, 1.04)	0.96
HbA1c (%)		
Age < 60 years	-0.03 (-0.09, 0.02)	0.18
Age ≥ 60 years	-0.20 (-0.30, -0.09)	< 0.01
hsCRP (mg/L)		
Age < 60 years	-0.34 (-0.74, 0.07)	0.10
Age ≥ 60 years	0.05 (-1.04, 1.14)	0.92
Uric acid (μmol/L)		
Age < 60 years	14.78 (9.15, 20.41)	< 0.01
Age ≥ 60 years	19.12 (8.38, 29.86)	< 0.01

[1] Data were presented as β coefficients (95% CI). Model 1 was a simple linear regression model. Model 2 was a multiple linear regression model and adjusted for potential confounders, including sex, BMI, urban index, region, education level, alcohol intake, smoking status,

physical activity, blood pressure, cholesterol, fiber, saturated fatty acids, monounsaturated fatty acids, polyunsaturated fatty acids, other fatty acids and total energy. CMRF, cardiometabolic risk factor; HbA1c, hemoglobin A1c; HDL-C, high-density lipoprotein cholesterol; HOMA-IR, homeostasis model assessment of insulin resistance; hsCRP, high-sensitive C-reactive protein; LDL-C, low-density lipoprotein cholesterol; TC, total cholesterol; TG, triglycerides.

Supplemental Material

Table S5. Associations between the animal to plant protein ratio and cardiometabolic risk factors according to region among 7886 Chinese adults who participated in China Health and Nutrition Survey 2009. [1]

Cardiometabolic risk factors	The animal to plant protein ratio	
	β (95% CI)	*p*
Lipid and lipoprotein profiles		
TG (mmol/L)		
Northern	0.06 (-0.08, 0.20)	0.40
Southern	0.02 (-0.07, 0.11)	0.66
TC (mmol/L)		
Northern	0.12 (0.02, 0.23)	0.02
Southern	0.09 (0.02, 0.16)	0.01
HDL-C (mmol/L)		
Northern	0.02 (-0.02, 0.06)	0.39
Southern	0 (-0.02, 0.03)	0.90
LDL-C (mmol/L)		
Northern	0.08 (-0.03, 0.19)	0.17
Southern	0.09 (0.03, 0.16)	0.01
Non-HDL-C (mmol/L)		
Northern	0.08 (-0.03, 0.20)	0.15
Southern	0.08 (0.01, 0.15)	0.03
TC:HDL-C		
Northern	0.04 (-0.09, 0.18)	0.53
Southern	0.04 (-0.04, 0.11)	0.38
LDL-C:HDL-C		
Northern	0.03 (-0.07, 0.13)	0.61
Southern	0.06 (0.01, 0.12)	0.03
Lipoprotein (a) (mg/L)		
Northern	1.19 (-15.69, 18.08)	0.89
Southern	8.62 (-2.49, 19.73)	0.13
Glucose homeostasis biomarkers		
Glucose (mmol/L)		
Northern	-0.15 (-0.30, 0)	0.05
Southern	-0.03 (-0.11, 0.05)	0.53
Insulin (μIU/mL)		
Northern	0.55 (-1.52, 2.62)	0.60
Southern	-0.75 (-2.19, 0.70)	0.31
HOMA-IR		
Northern	-0.01 (-0.74, 0.72)	0.98
Southern	-0.33 (-0.74, 0.08)	0.11
HbA1c (%)		
Northern	-0.15 (-0.24, -0.06)	< 0.01
Southern	-0.04 (-0.09, 0.02)	0.18
hsCRP (mg/L)		
Northern	-0.09 (-0.63, 0.46)	0.76
Southern	-0.28 (-0.85, 0.29)	0.34
Uric acid (μmol/L)		
Northern	24.82 (15.33, 34.31)	< 0.01
Southern	11.07 (4.82, 17.31)	< 0.01

[1] Data were presented as β coefficients (95% CI). Model 1 was a simple linear regression model. Model 2 was a multiple linear regression model and adjusted for potential confounders, including age, sex, BMI, urban index, education level, alcohol intake, smoking status, physical

activity, blood pressure, cholesterol, fiber, saturated fatty acids, monounsaturated fatty acids, polyunsaturated fatty acids, other fatty acids and total energy. CMRF, cardiometabolic risk factor; HbA1c, hemoglobin A1c; HDL-C, high-density lipoprotein cholesterol; HOMA-IR, homeostasis model assessment of insulin resistance; hsCRP, high-sensitive C-reactive protein; LDL-C, low-density lipoprotein cholesterol; TC, total cholesterol; TG, triglycerides.

Supplemental Material

Figure S1. Participant flow diagram.

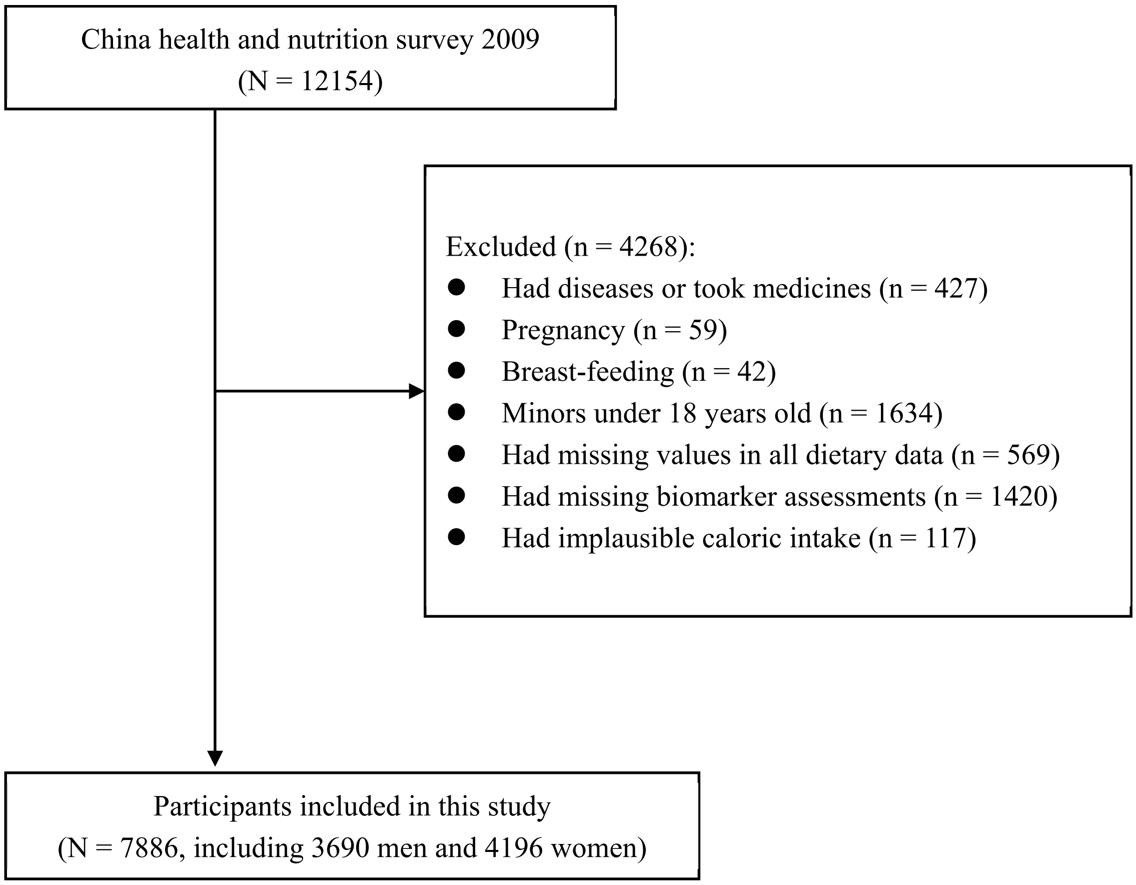

Supplement: Supplementary file 1 [file nutrients-13-00336-s001.pdf]
